# Selective Serial Multi-Antibody Biosensing with TOPAS Microstructured Polymer Optical Fibers

**DOI:** 10.3390/s130303242

**Published:** 2013-03-08

**Authors:** Grigoriy Emiliyanov, Poul E. Høiby, Lars H. Pedersen, Ole Bang

**Affiliations:** 1 DTU Fotonik, Department of Photonics Engineering, Technical University of Denmark, Ørsteds Plads, 2800 Kgs. Lyngby, Denmark; E-Mail: egrigoriy@hotmail.com; 2 DAKO Denmark A/S, Produktionsvej 42, DK-2600 Glostrup, Denmark; E-Mail: peh@exiqon.com; 3 Bioneer A/S, Kogle Allé 2, 2970 Hørsholm, Denmark; E-Mail: lap@bioneer.dk

**Keywords:** microstructured polymer optical fiber, fluorescence, antibodies

## Abstract

We have developed a fluorescence-based fiber-optical biosensor, which can selectively detect different antibodies in serial at preselected positions inside a single piece of fiber. The fiber is a microstructured polymer optical fiber fabricated from TOPAS cyclic olefin copolymer, which allows for UV activation of localized sensor layers inside the holes of the fiber. Serial fluorescence-based selective sensing of Cy3-labelled α-streptavidin and Cy5-labelled α-CRP antibodies is demonstrated.

## 1. Introduction

The development of fiber-optical biosensors is driven by the need for simple, rapid, and continuous *in situ* monitoring techniques for accurate detection of biomolecules in the biomedical, pharmaceutical, environmental, defense, bioprocessing, and food technology areas [1,2]. Fiber-optical biosensors combine two important scientific advances, the laser and the optical fiber. Low-loss light delivery, long interaction lengths, low fabrication costs, and the ability to not only excite target molecules, but also to capture the emitted light, are important advantages of optical fibers in the context of bio-sensing. Despite their non-planar geometry, fiber-optical biosensors may also be incorporated as active elements in a lab-on-a-chip configuration [3].

Here we consider biosensing with microstructured optical fibers (MOFs), which have a pattern of air holes running along the entire length of the fiber [4]. The optical properties of the fiber are primarily determined by the position, size, and shape of the air holes and MOFs exhibit a number of unique properties compared to conventional step-index fibers. The MOF can for example be endlessly single-mode [5] and it can be designed to guide either in a solid core through index guiding or in a hollow core through the photonic bandgap effect [6].

MOF biosensors have the advantage that bio-chemical reactions and definition of sensor layers can be performed inside the air holes. Biological samples may hence be probed by the optical field without removing the fiber coating and cladding, thus maintaining the robustness of the fiber. In addition, the sample volume can be minute (nanoliters), due to the small cladding holes. In general, biosensing with MOFs fall into two categories:

(1) Evanescent wave sensing of samples in the holes through the evanescent tail of the field propagating in the solid material. Typically this is done in the cladding holes of an index-guiding MOF.
(2) Sensing samples in the core of a hollow-core originally bandgap-guiding MOF with the fundamental mode then propagating with most of its power in the sample.

Evanescent wave sensing with MOFs was first proposed by Monro *et al.*, who numerically studied a MOF with a periodic triangular arrangement of cladding holes and showed that the overlap of the optical mode with the holes could be made sufficiently large if the pitch Λ (hole-to-hole separation) could be made small enough and the relative hole diameter d/Λ could be made small enough [7,8]. For Λ = 750 nm and d/Λ = 0.7 the overlap was for example 20% at a wavelength of 1,550 nm and decreasing with decreasing wavelength [7]. It was later shown by Du and co-workers that a 3-hole steering-wheel solid-core MOF design could give an even larger overlap of 29% at 1,500 nm and that the overlap increases when water was infiltrated in the fiber [9,10].

A large field overlap with the sample is essential for a good fiber-optical biosensor. Hollow-core bandgap MOFs with sample liquids in the core and the optical field guided in the core can have more than a 90% overlap and are thus potentially very attractive for biosensing, as proposed by Fini [11] and first demonstrated for gas sensing [12] and later for surface-enhanced Raman scattering (SERS) [13–15]. However, with liquid in the core the optical losses are typically very large in these biosensors, often selective filling is needed [11,14], and stringent requirements must be met on the periodicity of the holes in the cladding, whereas this is not the case for index guiding MOFs, where the hole-structure can even be random [16].

Evanescent-wave MOF sensors were first used for gas sensing [16,17] and in 2004 for sensing of fluorophore-labeled biomolecules in aqueous solutions [18]. The MOF biosensor used in [18] and later in the chip-based version [3] was special in that a hollow-core bandgap MOF was used, but filled uniformly in both core and cladding, while the fiber was excited in such a way, that the light was guided in all the tiny interstices between the holes. In this way a large overlap of the evanescent field with the holes was possible [3,18].

Evanescent wave MOF sensors have since been investigated intensively, with typical sensing modalities including absorption spectroscopy [3,18], fluorescence spectroscopy [19–25], Raman spectroscopy [9,26], surface-enhanced Raman spectroscopy [10,27–30], and resonance based sensing, such as long-period gratings (LPGs) [31–33], fiber Bragg gratings (FBGs) [32], fiber couplers [34], and four-wave mixing (FWM) [35].

In the above survey we have focused on applications of MOFs for biosensing and included also gas sensing. It is in this context important to note that in fact MOF refractive index sensors can be regarded as a biosensor also, in the sense that a MOF sensor capable of measuring the uniform refractive index of an analyte in the holes, will also be a sensitive biosensor capable of detecting biomolecular layers captured on the walls of the holes. MOF refractive index sensing has been demonstrated using bandgap edge tracking in hollow core [36,37] and solid-core [38,39] MOFs, in which the analyte should have a refractive index smaller and higher than the fiber host material, respectively, as well as with LPGs [32,40,41], FBGs [32,42], couplers [43–46], and FWM [47].

A major goal is to develop fiber-optical biosensors capable of performing rapid immunoassays, *i.e.*, detect several biological molecules in one or more measurements. Current multi-analyte fiber-optical biosensors, like the commercially available RAPTOR capable of detecting four analytes [48], detects in parallel with one fiber for each biomolecule. All MOF-based biosensors surveyed above have only dealt with the detection of a single biomolecule. Here we present the first fiber-optical biosensor capable of selectively measuring several analytes in series, inside a single MOF. This is achieved by using a polymer MOF made of the specific polymer TOPAS, whose properties are essential for the biosensor.

Polymer MOFs (or mPOF for microstructured polymer optical fiber) were first fabricated in 2001 [49] and are now routinely fabricated with a wealth of different hole structures [50], primarily in polymethylmethacrylate (PMMA). PMMA is very easy to functionalize with biomolecular layers and thus PMMA mPOFs are very appropriate for selective biosensing [19], where glasses require more steps to be functionalized with a biomolecular capture layer, as demonstrated for silica MOFs in 2006 [3,31] and softglass MOFs in 2008 [25]. Furthermore, polymers are much more biofriendly and thus suitable for *in vivo* sensing applications than glasses, and can even be made biodegradable [51].

TOPAS cyclic olefin copolymer (or just TOPAS) mPOFs were first fabricated in 2007 and demonstrated to be suitable for selective biosensing [21,22]. TOPAS has no monomers and its moisture absorption is hundred times lower than PMMA [52], which is why it is good for mPOF fiber drawing and why TOPAS MOFs are insensitive to humidity [53]. PMMA is so sensitive to humidity that PMMA mPOF FBGs have found application as humidity sensors [54]. Evidently, *in vivo* and many other sensing applications would benefit from the fiber material being humidity insensitive. TOPAS is photosensitive and thus TOPAS FBG sensors can be fabricated [53,55], and TOPAS is highly transparent in the THz regime, where PMMA is extremely lossy [56–58].

In contrast to PMMA, TOPAS is chemically inert and thus biomolecules are not easily immobilized onto it. However, commercially available anthraquinone (AQ) photolinkers (see www.exiqon.com) can attach to the TOPAS surface when activated by UV light and can subsequently accept sensor layers. This concept was used to develop the first flourescence-based TOPAS mPOF antibody biosensor, which had a reasonable selectivity [21,22]. Here we for the first time take full advantage of the chemical inertness of TOPAS and the AQ-linker technology and present the first fluorescence-based serial fiber-optical biosensor capable of selective detection of two different antibodies with a single fiber.

## 2. The Optical Fiber Sensor

**Figure 1.**
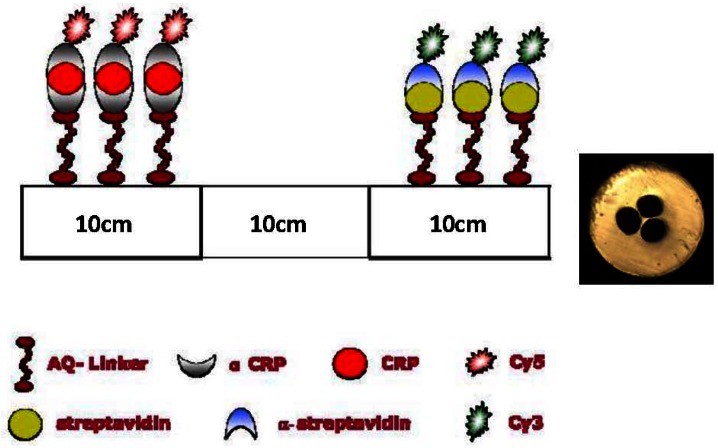
Biochemical immobilization procedure for multi-antibody detection. The illustration shows the ideal result of probing a sample with both antibodies, resulting in Cy3-labelled α-streptavidin captured in one 10 cm end and Cy5-labelled α-CRP captured in the other 10 cm end of the TOPAS mPOF (outer diameter 200 μm, hole diameter 40 μm, core diameter 12 μm), whose end facet is shown right.

In this first proof-of-concept we do not focus on optimizing the guiding properties of the mPOF. We therefore chose a simple 3-hole cladding structure with large holes in order to reduce the filling time. 3-hole MOFs have also been shown to allow a large overlap between the field and the holes [9,10] and been the ones used in several MOF biosensors [9,10,21,22,24,25,30,42]. The preform was fabricated from a TOPAS rod (TOPAS 8007 granules) with a diameter of 2.5 cm and a length of 7 cm. Three holes with a diameter of 2 mm were drilled into the preform, which was then drawn without pressure at 140 °C directly to the 200 μm in diameter fiber shown in Figure 1. For the loss of the TOPAS mPOF we refer to [59].

The concept of serial multi-analyte biosensing relies on defining different sections with different sensor layers inside a single TOPAS fiber. We have chosen to prepare a sensor that is able to detect the two antibodies: α-streptavidin and α-CRP. Using selective antigen-antibody binding, the immobilized sensing layers consist of the antigens streptavidin and CRP, respectively [19,21,22]. The preparation of the dual sensor layer is performed in a 30 cm long TOPAS mPOF (see [60] for the cleaving procedure) as follows: A 1.6 mg·L^−1^ AQ-Linker solution was flushed trough the fiber for 1 hour. Then the first 10 cm of the fiber was illuminated with UV light for 10 min. After a washing step with distilled water, 0.5 mg·mL^−1^ streptavidin in 0.1 M Na carbonate, pH 9.6 buffer was flushed through the fiber to be immobilized onto the UV-activated AQ linker molecules. After 1 hour of incubation, the holes were flushed for 3 minutes with phosphate buffered saline (PBS, 10 mM phosphate, pH 7.5). To eliminate the possibility of unblocked AQ-Linker molecules to bind non-specific nucleophiles at a later stage, an active-site blocking procedure was applied by injecting 10 mM ethanolamine in 100 mM sodium carbonate, pH 9.6 for 1 hour. After this a PBS wash was again performed. Thus the first 10 cm of the mPOF was ready for the selective capture of α-streptavidin antibodies.

The AQ-Linker solution was then re-applied through the fiber for 1 hour, but this time only the opposite 10 cm end of the fiber was illuminated with UV light. This step was followed by washing out surplus AQ-Linker with distilled water. The definition of a CRP layer in TOPAS mPOFs required two consecutive steps [19,21,22]. First α-CRP was bound to the surface (1 mg·mL^−1^ a-CRP in PBS for 30 min.) and then CRP was captured (0.040 mg·mL^−1^ CRP in PBS for 30 min). The blocking procedure described above was then applied for 1 hour and the fiber was subsequently washed with PBS. With this procedure we have thus obtained a 30 cm TOPAS mPOF biosensor, where the first 10 cm are prepared for the selective capture of α-streptavidin antibodies, and the final 10 cm prepared for the selective capture of α-CRP antibodies, as illustrated in Figure 1.

## 3. Flourescence Measurements

In our fluorescence detection scheme we use the standard fluorophore marker molecules Cy3 and Cy5. The Cy3 molecule has maximum absorption at 550 nm and maximum emission at 570 nm, whereas the Cy5 molecule has maximum absorption at 649 nm and maximum emission at 670 nm. We labeled α-streptavidin with Cy3 and α-CRP with Cy5. The fiber was then probed with a solution containing both α-streptavidin-Cy3 and α-CRP-Cy5 antibodies. After the probing the TOPAS mPOF was washed with PBS and water and then flushed with air for 3 min.

**Figure 2.**
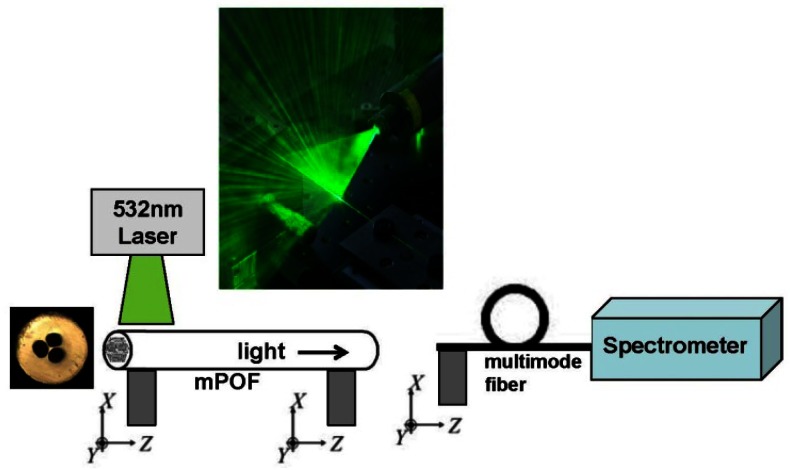
Experimental set-up for the optical characterization.

The optical characterization was carried out using a simple fluorescence setup shown in Figure 2, where the input 7 cm part of the mPOF is illuminated from the side by a 7 cm by 100 μm line-shaped beam from a 532 nm laser, as also used in [19,21,22]. Some of the light tunnels into the fiber core and is guided to the output end of the fiber, where it is detected with a spectrum analyzer. For this initial proof-of principle test the side-illumination works fine, while for future advance sensor characterizations conventional incoupling will be used. If the sensor functions as anticipated, then the 532 nm light should be able to excite the Cy3 fluorophores and the emission of Cy3 should just be able to excite the Cy5 fluorophores, allowing to detect the presence of both antibodies in one measurement.

**Figure 3.**
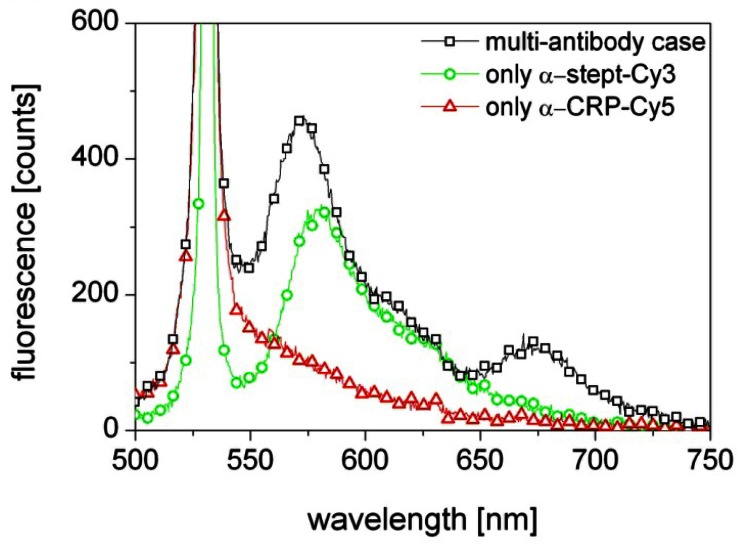
Flourescence measurements using side excitation with a 7 cm by 100 μm line-shaped beam from a 532 nm laser. TOPAS mPOF with both Streptavidin and α-CRP/CRP sensor sections, probed with both α-streptavidin-Cy3 and α-CRP-Cy5 (black squares); TOPAS mPOF with only a Streptavidin sensor section and probed with only α-streptavidin-Cy3 (green circles); TOPAS mPOF with only an α-CRP/CRP sensor layer and probed with only α-CRP-Cy5 (red triangles).

The results presented in Figure 3 (black squares) show indeed excitation of both Cy3 and Cy5, which means that the biosensor correctly detects the presence of both α-streptavidin and α-CRP antibodies in the probed solution. As the laser wavelength is close to the absorption line of the Cy3 flourophore the contribution from Cy3 is strong. In contrast, the 649 nm absorption line of Cy5 is far from the 570 nm emission line of Cy3 and the 532 nm laser wavelength and thus its excitation is weak. Most likely the Cy5 excitation is primarily due to the light emitted from Cy3 in the first section being guided through the fiber to the other end with the Cy5.

A control measurement was performed with a fiber activated with only a streptavidin sensor layer and probed with only α-streptavidin-Cy3. The result, shown in Figure 3 (green circles), confirms a strong excitation of Cy3 and thus the presence of α-streptavidin. Another control measurement was performed with a fiber activated with only an α-CRP/CRP sensor layer and probed with only α-CRP-Cy5. The result, shown in Figure 3 (red triangles), shows no excitation of Cy5, despite α-CRP-Cy5 knowingly being in the solution. This confirms that the Cy5 emission observed in the original experiment was indeed solely due to excitation by the light emitted by the Cy3 flourophores at 570 nm.

## 4. Discussion

The fact that the AQ-linker UV-activation technology could be used to define a localized sensor layer inside the holes of a TOPAS mPOF, was demonstrated in [21], in which we also showed epifluorescence microscope pictures of the 2 mm ends of the fiber. These pictures confirmed a much stronger fluorescence from the activated section of the fiber, but also revealed a weak fluorescence being present in the other non-activated end.

This means that our here presented dual-analyte sensor will have a challenge with “cross-talk” between the two sensor sections originating from a non-optimized UV irradiation in defining the sensor layers—a smaller dose might be sufficient, which could reduce the amount of UV light guided between sections. Another source of cross-talk could be that our applied biochemical procedure could allow AQ-linker molecules intended only for the 2nd section to attach to the sensor layer of the first section. Future work should therefore involve characterizing and eliminating this “cross-talk”. However, this investigation is out of the scope of the present proof-of-principle demonstration.

In conclusion, we have presented the first selective serial multi-analyte fluorescence-based fiber-optical biosensor, which is able to selectively detect more than one analyte inside a single fiber. The example we have presented is a serial dual-antibody biosensor able to detect α-streptavidin and α-CRP inside a single fiber, but the technique may be extended to more antibodies and to other biomolecules. The demonstrated serial selective multi-antibody detection derives its importance from the the spatial separation of the sensor layers made possible by using the polymer TOPAS, which provides the possibility for future selective and label-free multi-antibody biosensing by writing FBGs with different center wavelengths in the different spatially separated sections. Such label-free sensing would not be possible if the two sensor-layers were intermixed in one spatially homogeneous multi-antibody sensor layer.
